# Load Identification for a Cantilever Beam Based on Fiber Bragg Grating Sensors

**DOI:** 10.3390/s17081733

**Published:** 2017-07-28

**Authors:** Xuegang Song, Yuexin Zhang, Dakai Liang

**Affiliations:** 1 State Key Laboratory of Mechanics and Control of Mechanical Structures, Nanjing University of Aeronautics and Astronautics, 210000 Nanjing, China; sxg37068219890209@126.com; 2 Key Laboratory of Micro-Inertial Instrument and Advanced Navigation Technology, Ministry of Education, School of Instrument Science and Engineering, Southeast University, 210000 Nanjing, China; smileyuexin@163.com

**Keywords:** load identification, FBG sensors, Kalman filter, CKF, recursive least-squares algorithm

## Abstract

Load identification plays an important role in structural health monitoring, which aims at preventing structural failures. In order to identify load for linear systems and nonlinear systems, this paper presents methods to identify load for a cantilever beam based on dynamic strain measurement by Fiber Bragg Grating (FBG) sensors. For linear systems, the proposed inverse method consists of Kalman filter with no load terms and a linear estimator. For nonlinear systems, the proposed inverse method consists of cubature Kalman filter (CKF) with no load terms and a nonlinear estimator. In the process of load identification, the state equations of the beam structures are constructed by using the finite element method (FEM). Kalman filter or CKF is used to suppress noise. The residual innovation sequences, gain matrix, and innovation covariance generated by Kalman filter or CKF are used to identify a load. To prove the effectiveness of the proposed method**,** numerical simulations and experiments of the beam structures are employed and the results show that the method has an excellent performance.

## 1. Introduction

Load identification is an important research area in structural health monitoring [1,2,3,4]. For reliability and cost effectiveness in the design and analysis of structures, accurate identification of the location and magnitude of a load is desirable. The stresses of structures are a function of load, and knowledge of load can be helpful for design optimization and health of structures. For aeronautical and space systems, load identification is used to prevent structural failures. For bridge-vehicle systems, load identification is used to design, diagnose, and maintain bridges. By identifying the load, we can also reduce reliance on expensive and time-consuming experiments. In engineering applications, force measurement sensors cannot be directly installed sometimes due to the operating conditions. In this situation, it is helpful to use attached sensors such as strain gauges or fiber optic sensors to measure in-plane strains of the structures. FBG sensors show significant potential in engineering applications. Their light weight, accuracy, and high spatial resolution distinguish them from traditional strain gauges and make them applicable to a variety of aeroelastic systems such as unmanned aerial vehicles and flexible flying wings that have strict weight requirements. So this paper focuses on identifying a load by using FBG sensors.

Load identification algorithms [3,4,5,6,7] can be divided into time domain algorithms and frequency domain algorithms. Compared to frequency domain algorithms, time domain algorithms are much more valuable. In the study of time domain algorithms, Ma [8,9] proposes a method to identify the load for linear systems by measuring all nodal displacements and rotations. In his work, Kalman filter is used to suppress noise, then residual innovation sequences, gain matrix, and innovation covariance generated by Kalman filter are used to identify a load using a least-squares method. At the foundation of Ma’s work, Lin [10] proposes a method to identify a load for nonlinear systems with a linear estimator by combing the extended Kalman filter (EKF) with a least-squares method. The key to Lin’s method is using EKF to turn nonlinear systems to linear systems so as to use a linear estimator to identify the load. For now, Ma’s and Lin’s work is limited to theoretical research, and cannot be directly used in engineering applications as nodal displacements and rotations are not easily measured. At the same time, the system of load identification needs to stall many sensors, which is difficult to realize in engineering applications. To amend these flaws, this work focuses on identifying a load by applying FBG sensors. FBG sensors have great advantages than other types of sensors, which are immune to electromagnetic interference, small enough to be embedded into structures and easy to complete distributed measurement. The distributed FBG sensing network can also solve the difficulties of sensor installation. For Lin’s work, turning nonlinear systems to linear systems with EKF can cause errors, and may result in sub-optimal performance and divergence when dealing with strong nonlinear systems. To amend this flaw, this work proposes a new method that is based on CKF and a nonlinear estimator to identify a load for nonlinear systems. 

In engineering applications, structures are usually simplified as a linear beam or a nonlinear beam. So the paper uses a linear beam and a nonlinear beam as the model to verify the proposed method, respectively. The finite element method is used to construct dynamic model of the beam structure, and the strain values getting from FBG sensors are employed as observed values. For linear systems, Kalman filter is used to suppress noise, then residual innovation sequences, gain matrix, and innovation covariance generated by Kalman filter are used to identify a load by using a linear estimator. For nonlinear systems, CKF is used to suppress noise, then the residual innovation sequences, priori state estimate, gain matrix and innovation covariance generated by CKF are employed to identify a load by using a nonlinear estimator. To verify the accuracy of the identification method, the simulations and experiments of a linear system and a nonlinear system are employed, and the results show that the system based on FBG sensors has an excellent performance. 

## 2. Sensing Principle

In this paper, FBG sensing network is established to measure the responses of structures. The relationship between wavelength shift and strain in FBG is shown in Equation (1). From Equation (1), we can get strain value ε. Here ∆λ is wavelength shift of FBG; ε is FBG axial strain; α is thermal expansion coefficient of FBG; ξ is thermo-optical coefficient of FBG; ∆T is amount of temperature change; and $P_{e}$ is the effective photo-elastic coefficient of FBG [11].

(1) $$\Delta \lambda =\lambda [(1-P_{e})\varepsilon +(a+\xi )\Delta T]$$

The finite element method is used to construct the state-space model of a beam structure. The finite element model is considered to be a system of “2*n*”-degrees-of-freedom and we paste two FBG sensors on every element. We establish the relationship between strain values, nodal displacements and nodal rotations as follows:

(2) $$\left[\begin{array}{l} \varepsilon_{1}\\ \varepsilon_{2}\\ \vdots \\ \vdots \\ \varepsilon_{2n-1}\\ \varepsilon_{2n} \end{array}\right]=\left[\begin{array}{cccccccc} B_{1} & & & & & & & \\ B_{2} & & & & & & & \\ & & B_{3} & & & & & \\ & & B_{4} & & & & & \\ & & & & \ddots & & & \\ & & & & & \ddots & & \\ & & & & & & & B_{2n-1}\\ & & & & & & & B_{2n} \end{array}\right]\left[\begin{array}{l} w_{1}\\ \theta_{1}\\ w_{2}\\ \theta_{2}\\ \vdots \\ w_{n-1}\\ \theta_{n-1}\\ w_{n}\\ \theta_{n} \end{array}\right],$$

where $\delta ={\left[w_{i},\theta_{i,}w_{j},\theta_{j}\right]}^{T}$, $B(\xi )=\frac{1}{l^{2}}[-6+12\xi ,l(-4+6\xi ),6-12\xi ,l(-2+6\xi )]\times \frac{h}{2}$. B(ξ) is the shape function; l is the length of the beam element; ξ=x/l, *x* is the location of the FBG in element; *h* is the thickness of the beam; w is the nodal displacement; and θ is the nodal rotation.

## 3. Load Identification of a Linear Beam System

### 3.1. Identification Principle

There are three steps to identify the location and magnitude of a load. First, the state equation and measurement equation of the state-space model are discretized. Second, a Kalman filter is used to suppress noise. Finally, the residual innovation sequences, gain matrix, and innovation covariance generated by Kalman filter are used to identify a load. 

#### 3.1.1. Linear System Discretization

The vibration equation of the discrete cantilever beam can be written as follows:

(3) $$M\ddot{Y}(t)+C\dot{Y}(t)+KY(t)=F(t),$$

where M is the n×n mass matrix; C is the n×n damping matrix;  K is the n×n stiffness matrix; F(t) is the n×1 force vector; and $\ddot{Y}\left(t\right),$ $\dot{Y}\left(t\right)$, and Y(t) are the n×1 acceleration vector, velocity vector, and displacement vector, respectively. 

According to the second-order dynamic system and measuring principle, the state equation and measurement equation of the state-space model can be written as:

(4) $$\dot{X}(t)=AX(t)+BF(t)$$

(5) Z(t)=HX(t),

where:

$$A=\left[\begin{array}{cc} 0_{n\times n} & I_{n\times n}\\ -M^{-1}K & -M^{-1}C \end{array}\right],B=\left[\begin{array}{l} 0_{n\times n}\\ M^{-1} \end{array}\right],\;H=\left[\begin{array}{cccccccc} B_{1} & & & & & & & \\ B_{2} & & & & & & & \\ & & B_{3} & & & & & \\ & & B_{4} & & & & & \\ & & & & \ddots & & & \\ & & & & & \ddots & & \\ & & & & & & & B_{2n-1}\\ & & & & & & & B_{2n} \end{array}\right].$$

State value $X\left(t\right)\;=\;{\left[X_{1}\left(t\right),X_{2}\left(t\right),\ldots X_{2n-1}\left(t\right),X_{2n}\left(t\right)\right]}^{T}$, force value $F\left(t\right)={\left[F_{1},F_{2},F_{3},\ldots F_{n}\right]}^{T}$. H is the 2n×2n measurement matrix and Z(t) represents the strain vector. 

Equations (4) and (5) are discretized over time intervals of length  △t to become:

(6) X(k+1)=ΦX(k)+Γ(F(k)+w(k))

(7) Z(k)=HX(k)+v(k)

(8) Φ=exp(AΔt)

(9) $$\Gamma =\int_{(k-1)\Delta t}^{k\Delta t}\exp[A(k\Delta t-\tau )]Bd\tau ,$$

where X(k) represents the state vector; Φ represents the state transition matrix; Γ represents the input matrix; △t represents the sampling interval; and F(k) represents the load sequence. $E\left(w\left(k\right)\right)=0,\;E\left(w\left(k\right)w^{T}\left(j\right)\right)=Q\delta_{\mathrm{kj}}$. E(v(k))=0, $\;E\left(v\left(k\right)v^{T}\left(j\right)\right)={R\delta }_{\mathrm{kj}}$. $E\left[w\left(k\right)\right]=0,\;E\left[w\left(k\right)w^{T}\left(l\right)\right]=Q\left(k\right)\delta_{\mathrm{kl}},\;Q=Q_{w}I_{2n*2n}$, where vector *w*(k) represents the process white noise, *Q* represents the covariance matrix, and $\delta_{\mathrm{kl}}$ is the Kronecker deltas. E[v(k)]=0, $\;E\left[v\left(k\right)v^{T}\left(l\right)\right]=R\left(k\right)\delta_{\mathrm{kl}},\;R=R_{v}I_{2n*2n}$, where vector *v*(*k*) represents the measurement white noise, *R* represents noise covariance matrix, $R_{v}=\sigma^{2}$, *σ* is the standard deviation of the measurement noise. The vectors *w*(k) and *v*(k) are mutually uncorrelated.

#### 3.1.2. Kalman Filter

The equations of Kalman filter are as follows:

(10) $$\bar{X}(k/k-1)=\Phi \bar{X}(k-1/k-1)$$

(11) $$P(k/k-1)=\Phi P(k-1/k-1)\Phi^{T}+\Gamma Q\Gamma^{T}$$

(12) $$S(k)=HP(k/k-1)H^{T}+R$$

(13) $$K_{a}(k)=P(k/k-1)H^{T}S^{-1}(k)$$

(14) $$P(k/k)=[I-K_{a}(k)H]P(k/k-1)$$

(15) $$\bar{Z}(k)=Z(k)-H\bar{X}(k/k-1)$$

(16) $$\bar{X}(k/k)=\bar{X}(k/k-1)+K_{a}(k)\bar{Z}\left(k\right),$$

where $\bar{X}(k/k-1)$ and $\bar{X}(k/k)$ are state vectors; Φ is the state transition matrix; H is measurement matrix; P(k/k−1) and P(k/k) are the covariance matrices; $K_{a}(k)$ is gain matrix; $\bar{Z}\left(k\right)$ represents the innovation matrix.

#### 3.1.3. Linear Estimator

The residual innovation sequences, gain matrix, and innovation covariance generated by Kalman filter are employed to calculate load by using a least-squares algorithm. The detailed derivation of the identification method can be found in Appendix A. The simple equations of the linear estimator are as follows:

(17) $$B_{s}(k)=H[\Phi M_{s}(k-1)+I]\Gamma$$

(18) $$M_{s}(k)=[I-K_{a}(k)H][\Phi M_{s}(K-1)+I]$$

(19) $$K_{b}(k)=\gamma^{-1}P_{b}(k-1)B^{T}{}_{s}(k){\left[B_{s}(k)\gamma^{-1}P_{b}(k-1)B^{T}{}_{s}(k)+S(k)\right]}^{-1}$$

(20) $$P_{b}(k)=[I-K_{b}(k)B_{s}(k)]\gamma^{-1}P_{b}(k-1)$$

(21) $$\hat{F}(k)=\hat{F}(k-1)+K_{b}(k)[\bar{Z}(k)-B_{s}(k)\hat{F}(k-1)],$$

where $K_{a}\left(k\right)$ represents the Kalman gain matrix, $B_{s}\left(k\right)\;$ and $M_{s}\left(k\right)\;$ represent the sensitivity matrices, $\bar{Z}\left(k\right)$ represents the innovation matgrix, $K_{b}\left(k\right)\;$ represents the correction gain for updating $\hat{F}\left(k\right)$, $P_{b}\left(k\right)$ represents the error covariance of the estimated input vector, and $\hat{F}\left(k\right)$ represents the estimated input vector. The fading factor *γ* is used to compromise between the loss of estimation accuracy and the fast adaptive capability. In this study, *γ* is set to a constant value (i.e., 0.69). 

### 3.2. Numerical Simulations of a Linear Beam System

**Figure 1:**
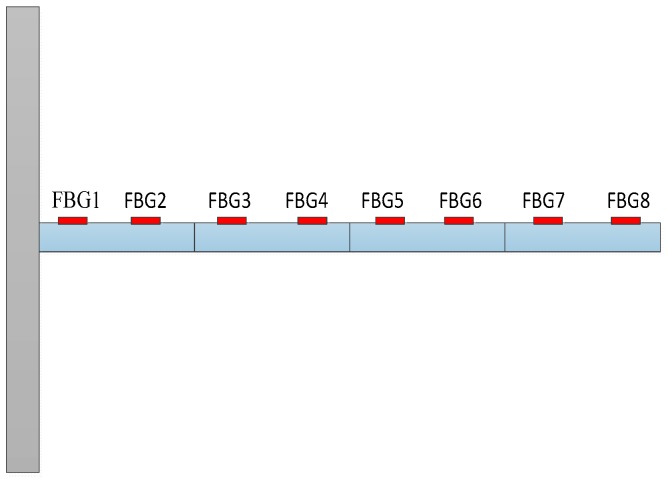
Beam model and FBG sensing network.

To verify the practicability and accuracy of the proposed method, numerical simulations of a cantilever beam are employed. The finite element model and the FBG sensing network of the cantilever beam are shown in Figure 1. The model parameters are given in Table 1. For the cantilever beam, the element mass matrix $M^{e}$, element stiffness matrix $K^{e}$, and proportional damping matrix C are as follows:

$$M^{e}=\frac{\rho Al}{3360}\left[\begin{array}{cccc} 624 & 44l & 216 & -26l\\ 44l & 4l^{2} & 26l & -3l^{2}\\ 216 & 26l & 624 & -44l\\ -26l & -3l^{2} & -44l & 4l^{2} \end{array}\right],\;K^{e}=\frac{4EI}{l^{3}}\left[\begin{array}{cccc} 24 & 6l & -24 & 6l\\ 6l & 2l^{2} & -6l & l^{2}\\ -24 & -6l & 24 & -6l\\ 6l & l^{2} & -6l & 2l^{2} \end{array}\right],\;C=\alpha M+\beta K$$

where *ρ* is the mass per unit length of the beam, l the length of the beam element, *E* the elastic modulus and *I* the moment of inertia of the cross-section, and *α* and *β* are constants with proper units. Considering a four-element beam, the global matrices *M* and *K* of the beam are obtained by assembling the matrices $M^{e}$ and $K^{e}$. Three types of load (sinusoidal, triangular, and rectangular) are considered in the simulations. 

**Figure 2:**
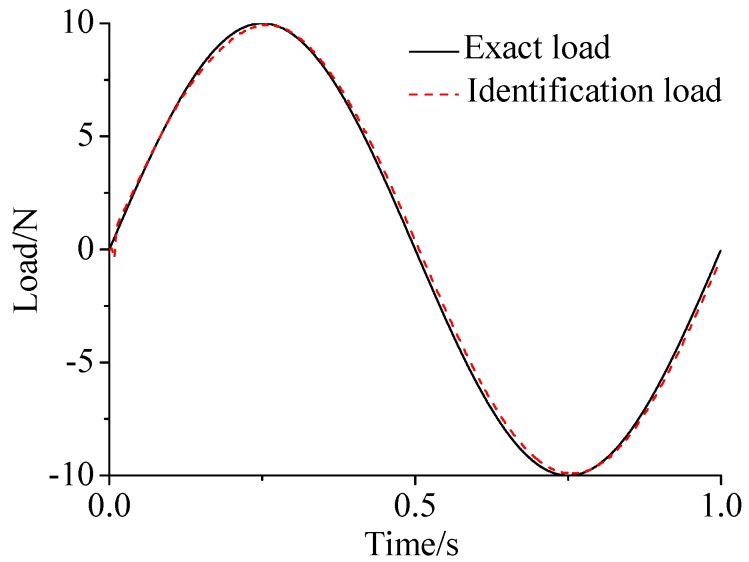
Identification result of the sinusoidal load.

**Figure 3:**
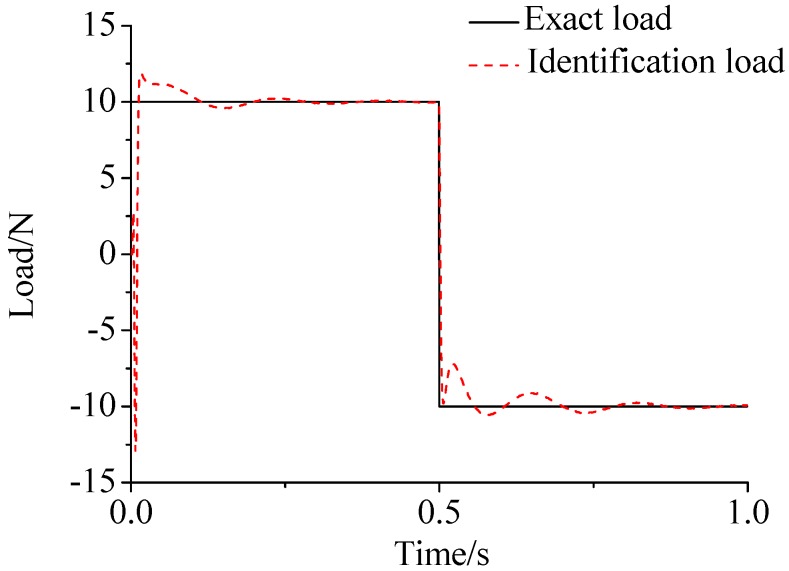
Identification result of the rectangular load.

**Figure 4:**
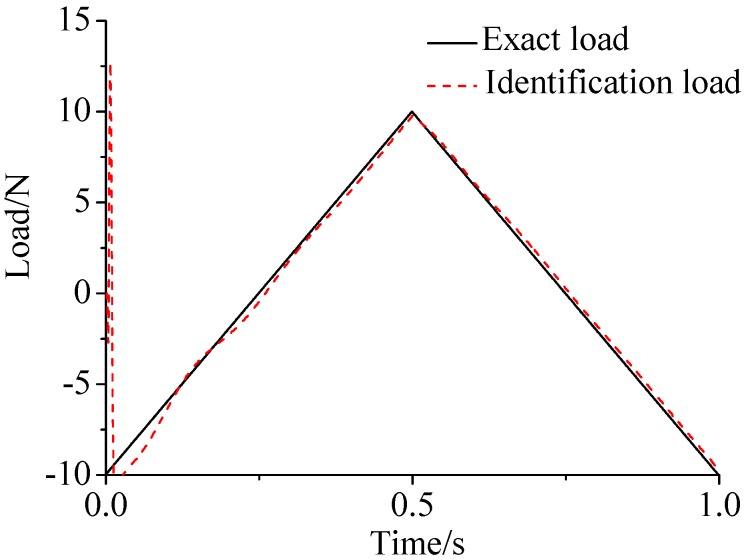
Identification result of the triangular load.

**Figure 5:**
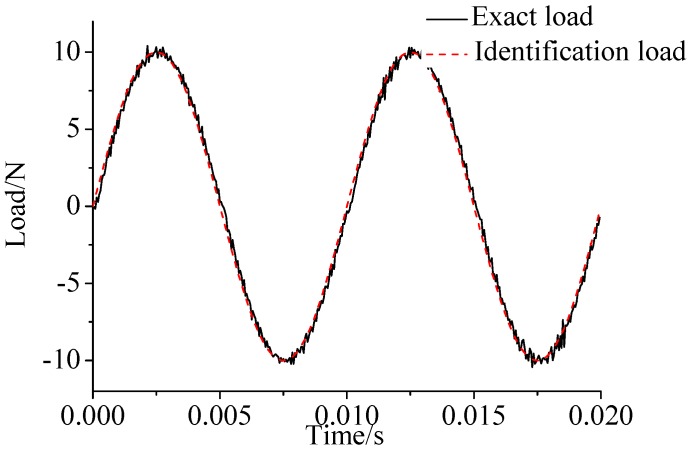
Identification result of the sinusoidal load.

**Figure 6:**
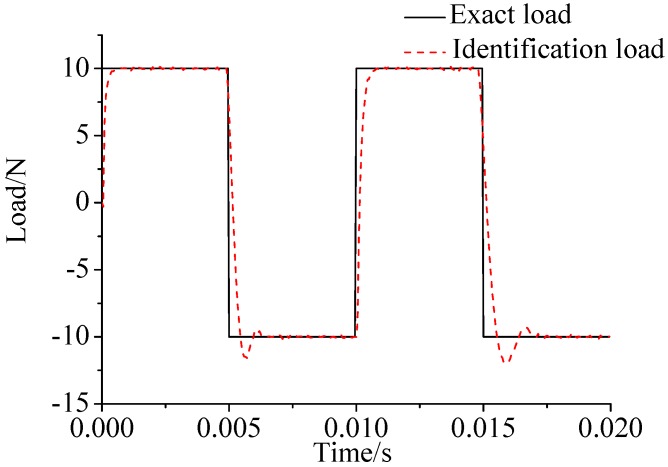
Identification result of the rectangular load.

**Figure 7:**
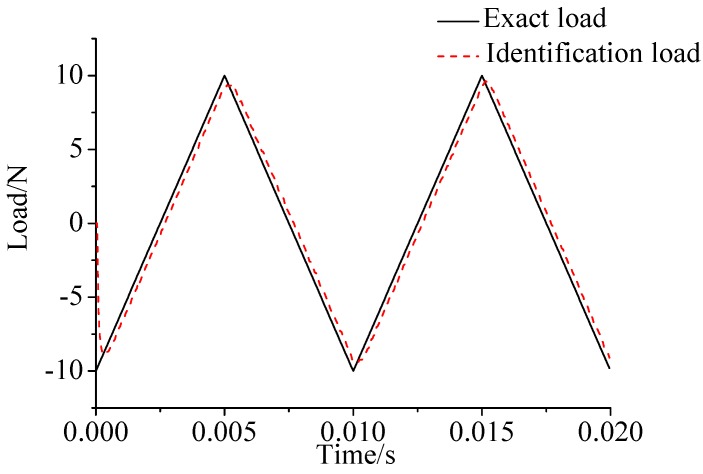
Identification result of the triangular load.

The system responses (strain values) with white noise are employed as the measurement values. The initial parameters of the estimation system are generally listed as follows: $x_{0}=zeros\left(16,1\right)$, $P_{1}=eye\left(16\right)$, $P_{2}=zeros\left(16\right)$, $M_{s}=200\times eye\left(16\right)$, $P_{b}=200\times eye\left(8\right),$ γ=0.69. The noise characteristic is set to $Q_{w}$ = 1 × e^−5^ and σ = 1 × e^−10^. The forcing frequency is set 1 Hz and 100 Hz, respectively. Load is applied at the end of the cantilever; the location and magnitude of load are identified from the strain responses. Figure 2, Figure 3 and Figure 4 plot the results of load identification with forcing frequency set 1 Hz and the sampling interval set ΔT = 0.01 s. Figure 5, Figure 6 and Figure 7 plot the results of load identification with forcing frequency set 100 Hz and the sampling interval set ΔT = 0.1 ms.

### 3.3. Experimental Verification of a Linear Beam System

**Figure 8:**
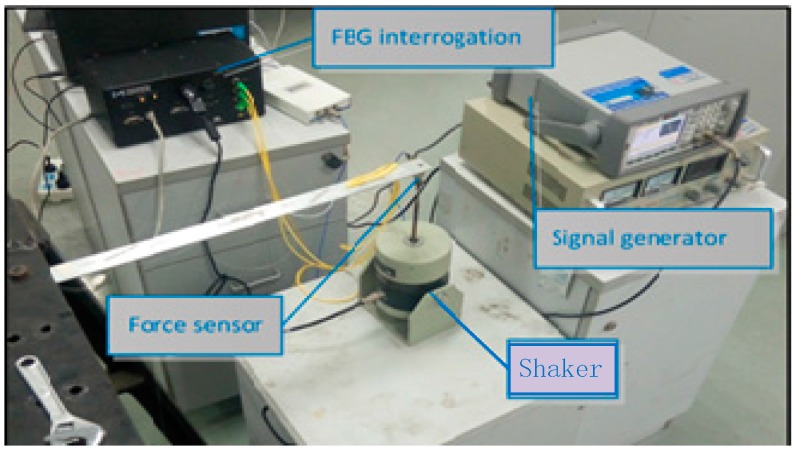
Layout of experiment.

**Figure 9:**
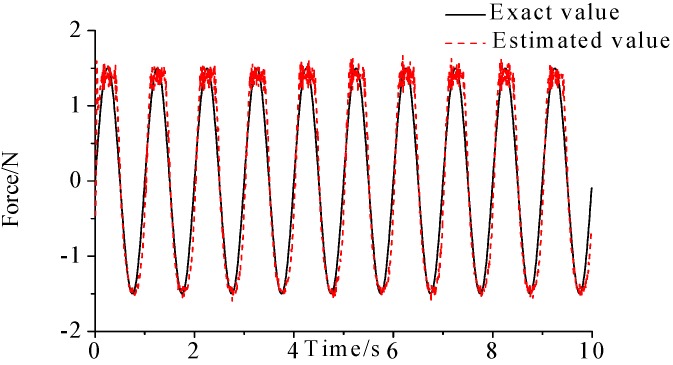
Identification result of the sinusoidal load.

The experiment is employed to verify the practicability and accuracy of the method, and the laboratory tests are performed on a simple support cantilever. Six FBG strain sensors are attached at the surface of the beam along its center line, to measure the axial dynamic strains, as shown in Figure 8. The distance between two consecutive sensors is about 9.15 cm. FBG interrogation system (SM130) is used for measuring the dynamic strains, and an electrodynamic shaker is employed for the excitation. The excitation point is at the end of the cantilever and a force sensor is also used at this location to measure the input force. The beam is excited with periodic sinusoidal signals, and the magnitude and location of the load are identified simultaneously from the dynamic strains. In the process, the measured force is used as exact value to verify the practicability of the proposed method. A NI cDAQ-9174 module and LABVIEW software are used to acquire signal. Parameters of the beam are: elastic modulus $=6.89\times 10^{10}\;\left(N/m^{2}\right),\;\mathrm{density}$ $\rho =2.69\times 10^{3}\;\left(\mathrm{kg}/m^{3}\right)$, beam length l=0.48 m, the cross section S=0.03 m×0.003 m. The damping matrix C is set as: C=0.01×M+0.02×K. Sampling frequency is set as 100 Hz, and experimental time is 10 s. The layout of the experiment is presented in Figure 8 and the identification results are plotted in Figure 9. 

## 4. Load Identification of a Nonlinear Beam System

### 4.1. Identification Principle

There are three steps to identify a load. First, the state equation and measurement equation of the state-space model are discretized. Second, a Cubature Kalman filter is used to suppress noise. Finally, the residual innovation sequences, a priori state estimate, gain matrix, and innovation covariance generated by CKF are used to identify the load. 

#### 4.1.1. Nonlinear System Discretization

The vibration equation of the nonlinear discrete beam can be written as follows:

(22) $$M\ddot{Y}(t)+C\dot{Y}(t)+K(Y)Y(t)=F(t),$$

where M is the n×n mass matrix; C is the n×n damping matrix; K is the n×n stiffness matrix and is varying with displacement vector; F(t) is the n×1 force vector; and $\ddot{Y}\left(t\right),$ $\dot{Y}\left(t\right)$, and Y(t) are the n×1 acceleration vector, velocity vector, and displacement vector, respectively. 

According to the second-order dynamic system and measuring principle, the state equation and measurement equation of the state-space model can be written as:

(23) $$\dot{X}(t)=f(X(t))+BF(t)$$

(24) Z(t)=HX(t),

where:

$$B=\left[\begin{array}{l} 0_{n\times n}\\ M^{-1} \end{array}\right],\;H=\left[\begin{array}{cccccccc} B_{1} & & & & & & & \\ B_{2} & & & & & & & \\ & & B_{3} & & & & & \\ & & B_{4} & & & & & \\ & & & & \ddots & & & \\ & & & & & \ddots & & \\ & & & & & & & B_{2n-1}\\ & & & & & & & B_{2n} \end{array}\right]$$

State value $X\left(t\right)={\left[X_{1}\left(t\right),X_{2}\left(t\right),\ldots X_{2n-1}\left(t\right),X_{2n}\left(t\right)\right]}^{T}$, force value $F\left(t\right)={\left[F_{1},F_{2},F_{3},\ldots F_{n}\right]}^{T}$. *f*(·) is a nonlinear function with respect to *X.* H is the 2n∗2n measurement matrix and Z(t) represents the strain vector. 

Discretize the Equations (23) and (24), and the discrete model can be described by:

(25) $$\left\{ \begin{array}{l} X_{k}=f(X_{k-1},F_{k-1})+w_{k}\\ Z_{k}=h(X_{k})+v_{k} \end{array}\right..$$

$X_{k}$ is state vector; $Z_{k}$ is measurement vector; *f*(·) and *h*(·) are a nonlinear functions. $E\left[w_{k}\right]=0,\;E\left[w_{k}w_{l}^{T}\right]=Q\delta_{kl},\;Q=Q_{w}I_{2n*2n}$, where vector $w_{k}$ represents the process white noise, *Q* represents covariance matrix and $\delta_{kl}$ is the Kronecker deltas. $E\left[v_{k}\right]=0$,$\;E\left[v_{k}v_{l}^{T}\right]=R\delta_{kl},\;R=R_{v}I_{2n*2n}$, where vector $v_{k}$ represents the measurement white noise, *R* represents noise covariance matrix, $R_{v}=\sigma^{2}$, *σ* is the standard deviation of the measurement noise. The vectors $w_{k}$ and $v_{k}$ are mutually uncorrelated. 

#### 4.1.2. Cubature Kalman Filter

Initialization: Initialize the filter by setting the initial state and covariance matrix [12,13]:

(26) $${\hat{X}}_{0/0}=E\left[X_{0/0}\right]$$

(27) $$P_{0/0}=E\left[\left(X_{0/0}-{\hat{X}}_{0/0}\right){\left(X_{0/0}-{\hat{X}}_{0/0}\right)}^{T}\right]$$

${\hat{X}}_{0/0}$ is the initial state vector, and $P_{0/0}$ is the initial covariance matrix.

Time update:

(1) Calculate the cubature points:
  $$S_{k-1/k-1}=chol\left(P_{k-1/k-1}\right)$$
  (28) $$X_{i,k-1/k-1}=S_{k-1/k-1}\xi_{i}+{\hat{X}}_{k-1/k-1},\;i=1,2,\ldots ,m,$$
  where ${\hat{X}}_{i,k-1/k-1}$ is the prior estimated state. $\xi_{i}=\sqrt{\frac{m}{2}}{\left[1\right]}_{i}$, ${\left[1\right]}_{i}$ is the *i*th column of the matrix [*I* (−1)*I*].
(2) Calculate the propagated cubature points:
  (29) $$X_{i,k/k-1}^{*}=f\left(X_{i,k-1/k-1},k-1\right).$$
(3) Calculate the predicted state and covariance matrix:
  (30) $${\hat{X}}_{k/k-1}=\frac{1}{m}\sum_{i=1}^{m}X_{i,k/k-1}^{*}$$
  (31) $$P_{k/k-1}=\frac{1}{m}\sum_{i=1}^{m}X_{i,k/k-1}^{*}X_{i,k/k-1}^{*T}-{\hat{X}}_{k/k-1}{\hat{X}}_{k/k-1}^{T}+{\hat{Q}}_{k-1}.$$

Measurement update:

(1) Calculate the cubature points:
  (32) $$S_{k/k-1}^{}=chol\left(P_{k/k-1}\right)$$
  (33) $$X_{i,k/k-1}=S_{k/k-1}\xi_{i}+{\hat{X}}_{k/k-1}.$$
(2) Calculate the propagated cubature points:
  (34) $$Y_{i,k/k-1}=h\left(X_{i,k/k-1}\right).$$
(3) Calculate the predicted state:
  (35) $${\hat{Z}}_{k/k-1}=\frac{1}{m}\sum_{i=1}^{m}Y_{i,k/k-1}.$$
(4) Calculate the innovation covariance matrix:
  (36) $$P_{zz,k/k-1}=\frac{1}{m}\sum_{i=1}^{m}Y_{i,k/k-1}Y_{i,k/k-1}^{T}-{\hat{Z}}_{k/k-1}{\hat{Z}}_{k/k-1}^{T}+{\hat{R}}_{k}.$$
(5) Calculate the cross-covariance matrix:
  (37) $$P_{xz,k/k-1}=\frac{1}{m}\sum_{i=1}^{m}X_{i,k/k-1}Y_{i,k/k-1}^{T}-{\hat{X}}_{k/k-1}{\hat{Z}}_{k/k-1}^{T}.$$
(6) Calculate the Kalman gain:
  (38) $$K_{k}=P_{xz,k/k-1}P_{zz,k/k-1}^{-1}.$$
(7) Calculate the updated state:
  (39) $${\bar{Z}}_{k}=Z_{k}-{\hat{Z}}_{k/k-1}$$
  (40) $${\hat{X}}_{k/k}={\hat{X}}_{k/k-1}+K_{k}{\bar{Z}}_{k}.$$
(8) Calculate the error covariance:
  (41) $$P_{k/k}=P_{k/k-1}-K_{k}P_{zz,k/k-1}K_{k}^{T}.$$

#### 4.1.3. The Nonlinear Estimator

Applying the residual innovation sequences, priori state estimate, gain matrix and innovation covariance generated by CKF, the location and magnitude of load can be identified by using a recursive least-squares algorithm from the response values (displacement, velocity, or acceleration). In the process, the first-order Taylor series expansion is used to around the estimated state value ${\hat{X}}_{k/k-1}$. The detailed derivation of the estimation method can be found in Appendix B. The simple equations of the least-squares estimator are as follows:

(42) $$\Phi_{k}=\partial f({\hat{X}}_{k/k-1})/\partial X$$

(43) $$\Gamma_{k}=\partial f({\hat{X}}_{k/k-1})/\partial F$$

(44) $$H_{k}=\partial h({\hat{X}}_{k/k-1})/\partial X$$

(45) $$B_{s}(k)=H_{k}[\Phi_{k}M_{s}(k-1)+I]\Gamma_{k}$$

(46) $$M_{s}(k)=[I-K_{k}H_{k}][\Phi_{k}M_{s}(k-1)+I]$$

(47) $$K_{b}(k)=\gamma^{-1}P_{b}(k-1)B_{s}{}^{T}(k){\left[B_{s}(k)\gamma^{-1}P_{b}(k-1)B_{s}{}^{T}(k)+P_{zz,k/k-1}\right]}^{-1}$$

(48) $$P_{b}(k)=[1-K_{b}(b)B_{s}(k)]\gamma^{-1}P_{b}(k-1)$$

(49) $$\hat{F}(k)=\hat{F}(k-1)+K_{b}(k)[{\bar{Z}}_{k}-B_{s}(k)\hat{F}(k-1)],$$

where *f*(·) and *h*(·) represent nonlinear functions of the discrete system; $P_{zz,k/k-1}$ represents the innovation covariance matrix; $K_{k}$ represents the gain matrix; $B_{s}\left(k\right)\;$ and $M_{s}\left(k\right)\;$ represent the sensitivity matrices; ${\bar{Z}}_{k}$ represents the innovation matrix; $K_{b}\left(k\right)\;$ represents the correction gain for updating $\hat{F}\left(k\right)$; $P_{b}\left(k\right)$ represents the error covariance; and $\hat{F}\left(k\right)$ represents the estimated input vector; *γ* is a fading factor. 

### 4.2. Numerical Simulations of a Nonlinear Beam System

**Figure 10:**
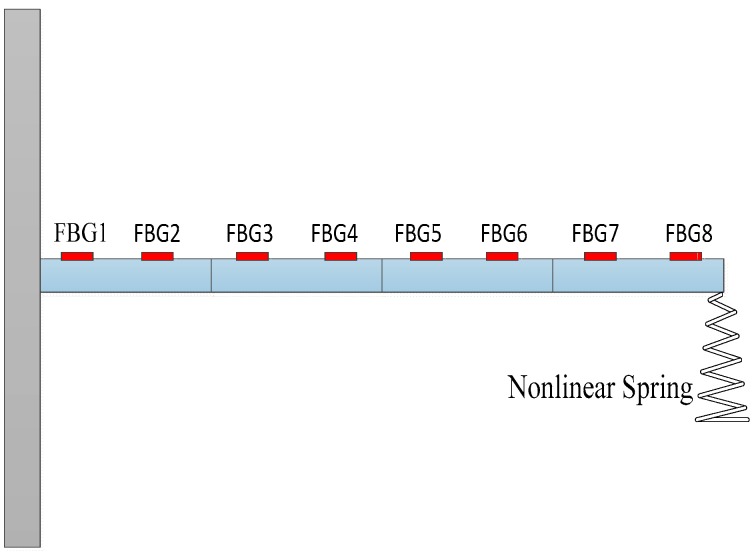
Beam model and FBG sensing network.

**Figure 11:**
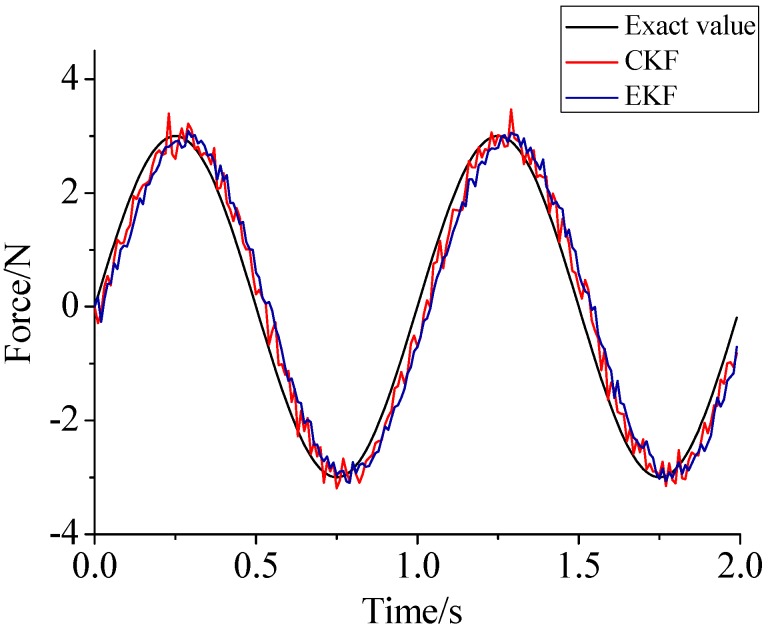
Identification result of the sinusoidal load.

**Figure 12:**
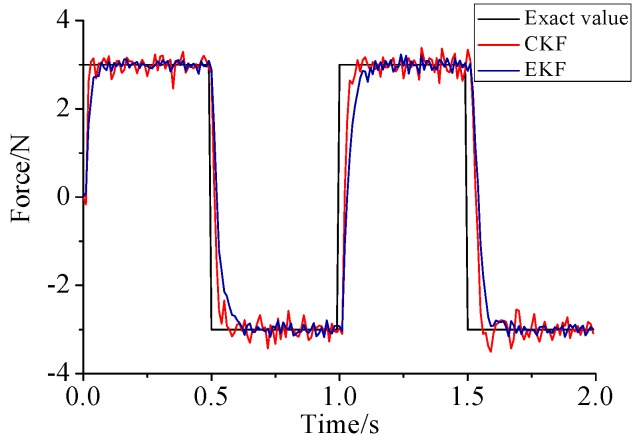
Identification result of the rectangular load.

**Figure 13:**
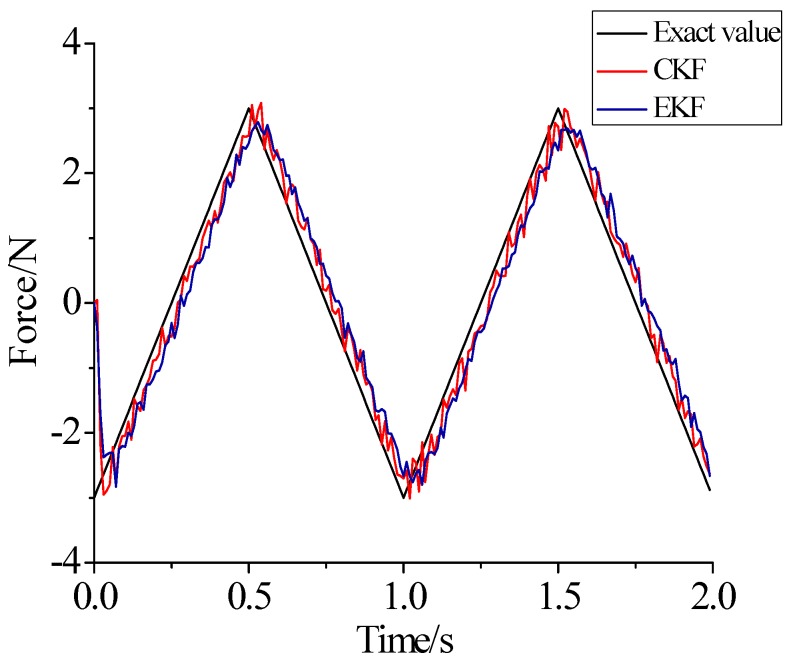
Identification result of the triangular load.

To verify the practicability and accuracy of load identification in nonlinear systems, numerical simulations of a cantilever beam that is constrained by a nonlinear spring at the end node are employed. The finite element model is the same as the model in Figure 10, and the model parameters are the same as in Table 1. Considering a four-element beam, the sampling interval is set as ΔT = 0.01 s. The system responses (strain values) with white noise are employed as the measurement values. Load is applied at the end of the cantilever; the location and magnitude of load are identified from the strain responses. The noise characteristic is set to $Q_{w}$ = 1 × e^−3^ and σ = 1 × e^−6^. The initial parameters of the estimation system are generally listed as follows: $x_{0}=zeros\left(16,1\right)$, $P_{1}=eye\left(16\right)$, $P_{2}=zeros\left(16\right)$, $M_{s}=200\times eye\left(16\right)$, $P_{b}=200\times eye\left(8\right),$ γ=0.69. For the beam model, sinusoidal load, rectangular load, and triangular load are employed. In order to contrast with Lin’s method, Figure 11, Figure 12 and Figure 13 plot the identified results based on EKF and the results based on CKF. 

### 4.3. Experimental Verification of a Nonlinear Beam System

**Figure 14:**
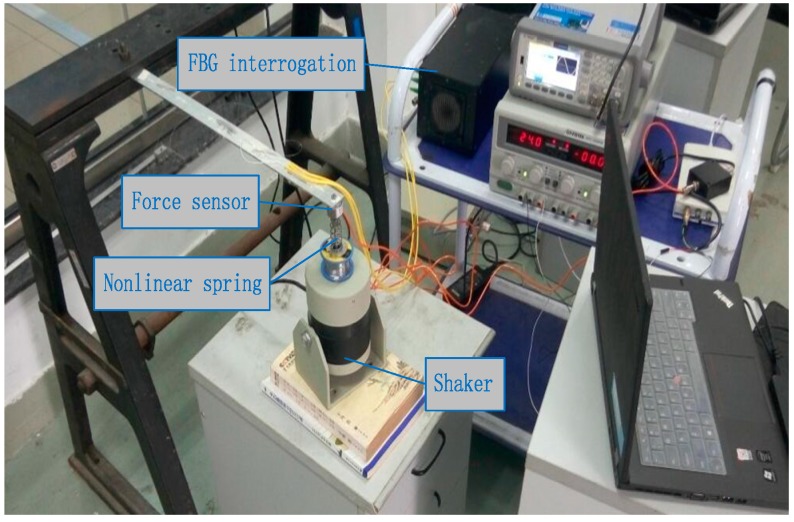
Layout of experiment.

**Figure 15:**
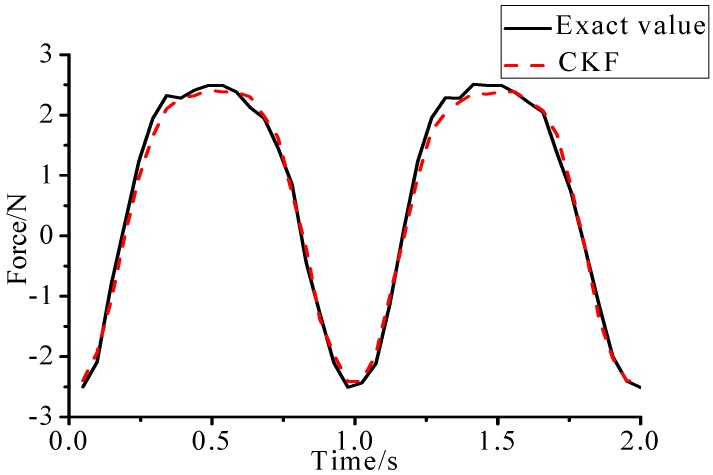
Identification result of experiment.

The experiment is employed to verify the practicability and accuracy of the identification method. Considering a linear three-element beam with a nonlinear spring stalled at the end nodal, six FBG strain sensors are attached at the surface of the beam along its center line, to measure the axial dynamic strains, as shown in Figure 14. The distance between two consecutive sensors is about 9.15 cm. FBG interrogation system (SM130) is used for measuring the dynamic strains, and an electrodynamic shaker is employed for the excitation. The excitation point coincided with a nonlinear spring is at the end of the cantilever and a force sensor is also used at this location to measure the input force. The beam is excited with periodic sinusoidal signals, and the magnitude and location of load are identified simultaneously from the dynamic strains. In the process, the measured force is used as exact value to verify the practicability of the proposed method. A NI cDAQ-9174 module and LABVIEW software are used to acquire signal. Parameters of the beam are: elastic modulus $=6.89\times 10^{10\;}\left(N/m^{2}\right),\;\mathrm{density}$ $\rho =2.69\times 10^{3}\;\left(\mathrm{kg}/m^{3}\right)$, beam length l=0.48 m, the cross section S=0.03 m×0.003 m. The damping matrix C is set as: C=0.01×M+0.02×K. Sampling frequency is set as 100 Hz, and experimental time is 2 s. The layout of experiment is presented in Figure 14 and the identification result is plotted in Figure 15. 

## 5. Discussion

(1) The proposed method of load identification is a recursive method that only needs the recent measurement values and the previously identified values to be kept in storage. This characteristic can save considerable memory and greatly decrease the system burden. The proposed method is based on a Kalman filter, and this can be helpful to control the beam structure by using optimal control theory after identifying the load.
(2) As illustrated in Figure 2, Figure 3, Figure 4, Figure 5, Figure 6 and Figure 7, Figure 11, Figure 12 and Figure 13, the identified load rapidly converges to the exact load. The identification performance of the sinusoidal load is better than that of the triangular load and rectangular load. As illustrated in Figure 9 and Figure 15, experimental results show that the load identification system based on FBG sensors has a good performance.
(3) The proposed method of load identification is based on a Kalman filter. As a Kalman filter can only be used to estimate continuous signal and cannot be used to estimate a random signal, the proposed method cannot be used to identify a random load. The identification results show a little delay between the exact load and the identified load, but we can apply iterative algorithms to decrease the delay. The identified load rapidly converges to the exact load, but with large initial estimation errors. To improve the performance of the initial estimation, the initial values of *P* and $P_{b}$ should be set to large values.

## 6. Conclusions

In order to identify the load of both linear beam systems and nonlinear beam systems, real-time methods based on FBG sensors are presented. The finite element method is used to construct a dynamic model of the beam structure, and the strain values obtained from FBG sensors are employed as observed values to identify the location and magnitude of the load. The proposed method is established on the foundation of a Kalman filter, which can be helpful to control the beam structure by using optimal control theory after load identification. At the same time, the proposed methods can identify a load accurately and solve the difficulty of sensor installation. Contrast this with Lin’s method, which is based on EKF. The method based on CKF has a better performance. This research has great value in engineering applications, and future studies will focus on the applications in aircraft structures.

## Figures and Tables

**Table 1:** Model parameters.

Bernoulli–Euler Beam	
Material	1540
Density (kg/ $m^{3}$)	2690
Elastic modulus (GPa)	68.9
Length (m)	0.64
Width (m)	0.03
Height (m)	0.003
Units number	4

## Appendix A

The discrete system is given below:

(A1) $$X_{k}=\Phi X_{k-1}+\Gamma F_{k-1}+w_{k}$$

(A2) $$Z_{k}=HX_{k}+v_{k}.$$

The posteriori state estimate without exciting force:

(A3) $${\bar{X}}_{k}=\Phi {\bar{X}}_{k-1}+K_{k}[Z_{k}-H\Phi {\bar{X}}_{k-1}].$$

The posteriori state estimate with exciting force:

(A4) $${\hat{X}}_{k}=\Phi {\hat{X}}_{k-1}+\Gamma F_{k-1}+K_{k}[Z_{k}-H\Phi {\hat{X}}_{k-1}-H\Gamma F_{k-1}],$$

where $K_{k}$ is obtained from KF.

Define the difference of the two posteriori state estimates as follows:

(A5) $$\begin{array}{ll} \Delta X_{k} & ={\hat{X}}_{k}-{\bar{X}}_{k}\\ & =(I-K_{k}H)\Phi ({\hat{X}}_{k-1}-{\bar{X}}_{k-1})+(I-K_{k}H)\Gamma F_{k-1} \end{array}.$$

Assuming the exciting force begins with time $t_{n}$, then:

1. *k* < *n*. ${\hat{X}}_{k-1}-{\bar{X}}_{k-1}$ = 0. $F_{k-1}$ = 0, so $\Delta X_{k}$ = 0
2. *k* = *n*. ${\hat{X}}_{k-1}-{\bar{X}}_{k-1}$ = 0. $F_{k-1}$ = 0, so $\Delta X_{k}$ = 0
3. *k* > *n*. ${\hat{X}}_{k-1}-{\bar{X}}_{k-1}=\Delta X_{k-1}$, so
  (A6) $$\begin{array}{ll} \Delta X_{k} & =(I-K_{k}H)\Phi ({\hat{X}}_{k-1}-{\bar{X}}_{k-1})+(I-K_{k}H)\Gamma F_{k-1}\\ & =(I-K_{k}H)(\Phi \Delta X_{k-1}+\Gamma F_{k-1}) \end{array}.$$

In summary, we get:

(A7) $$\Delta X_{k}=\left\{ \begin{array}{ll} 0 & k\leq n\\ \left(I-K_{k}H)(\Phi \Delta X_{k-1}+\Gamma F_{k-1}\right) & k>n \end{array}\right..$$

At time $t_{n+1}$, Equation (A7) becomes:

(A8) $$\Delta X_{n+1}=(I-K_{n+1}H){\left(\Phi \Delta X_{n}+\Gamma F_{n}\right)}_{}.$$

From Equation (A7) we know that $\Delta X_{n}$ = 0, so Equation (A8) becomes:

(A9) $$\Delta X_{n+1}=(I-K_{n+1}H)\Gamma F_{n}.$$

Define $M_{n+1}=I-K_{n+1}H$.

Then Equation (A9) becomes:

(A10) $$\Delta X_{n+1}=M_{n+1}\Gamma F_{n}.$$

From Equations (A7) and (A10), for *k* > *n*, we have:

(A11) $$\Delta X_{k}=(I-K_{k}H)(\Phi \Delta X_{k-1}+\Gamma F_{k-1}).$$

Ignoring $\Phi \Delta X_{k-1}$, Equation (A11) becomes:

(A12) $$\Delta X_{k}=M_{k}\Gamma F_{k-1}.$$

From Equations (A11) and (A12), we have:

$$\begin{array}{ll} M_{k}\Gamma F_{k-1} & =(I-K_{k}H)(\Phi \Delta X_{k-1}+\Gamma F_{k-1})\\ & =(I-K_{k}H)\Phi M_{k-1}\Gamma F_{k-2}+(I-K_{k}H)\Gamma F_{k-1} \end{array}.$$

Assume $F_{k-1}$ = $F_{k-2}$, then:

(A13) $$M_{k}=(I-K_{k}H)(\Phi M_{k-1}+I).$$

From Equations (A12) and (A13), we have:

(A14) $${\hat{X}}_{k}={\bar{X}}_{k}+M_{k}\Gamma F_{k-1}{}_{},$$

where:

$$M_{k}=\left\{ \begin{array}{ll} 0 & k\leq n\\ \left(I-K_{k}H)(\Phi M_{k-1}+I\right) & k>n \end{array}\right..$$

The observed value of the residual sequence with exciting force can be described as:

(A15) $${\hat{Z}}_{k}=Z_{k}-H[\Phi {\hat{X}}_{k-1}+\Gamma F_{k-1}].$$

The observed value of the residual sequence without exciting force can be described as:

(A16) $${\bar{Z}}_{k}=Z_{k}-H\Phi {\bar{X}}_{k-1}.$$

For different values of *k*, we have:

1. *k* < *n*. $F_{k-1}$ = 0, ${\bar{Z}}_{k}={\hat{Z}}_{k}$
2. *k* = *n*. $F_{k-1}$ = 0, ${\bar{Z}}_{k}={\hat{Z}}_{k}$
3. *k* > *n*. $F_{k-1}\neq 0$, ${\bar{Z}}_{k}\neq {\hat{Z}}_{k}$
  $$\begin{array}{ll} {\bar{Z}}_{k}-{\hat{Z}}_{k} & =H\Phi ({\hat{X}}_{k-1}-{\bar{X}}_{k-1})+H\Gamma F\\ & =H(\Phi M_{k-1}+I)\Gamma F \end{array}.$$

In summary, we get:

(A17) $${\bar{Z}}_{k}=\left\{ \begin{array}{ll} {\hat{Z}}_{k} & k\leq n\\ {\hat{Z}}_{k}+B_{k}F & k>n \end{array}\right.,$$

where:

$$B_{k}=H(\Phi M_{k-1}+I)\Gamma .$$

For *k* = *n* + 1, *n* + 2, ... *n* + *l*. we have:

(A18) Y=ψF+ε,

where:

$$Y(N)=\left[\begin{array}{l} {\bar{Z}}_{n+1}\\ {\bar{Z}}_{n+2}\\ \vdots \\ {\bar{Z}}_{n+l} \end{array}\right],\varepsilon (N)=\left[\begin{array}{l} {\hat{Z}}_{n+1}\\ {\hat{Z}}_{n+2}\\ \vdots \\ {\hat{Z}}_{n+l} \end{array}\right],\psi (N)=\left[\begin{array}{l} B(n+1)\\ B(n+2)\\ \vdots \\ B(n+l) \end{array}\right]=\left[\begin{array}{l} H\Gamma \\ H(\Phi M_{n+1}+I)\Gamma \\ \vdots \\ H(\Phi M_{n+l-1}+I)\Gamma \end{array}\right],M_{n+l}=\left\{ \begin{array}{ll} 0 & l=0\\ \left(I-K_{n+l}H)[\Phi M_{n+l-1}+I\right] & l>0 \end{array}\right..$$

Assume $E[\hat{z}(k){\hat{z}}^{T}(k)]=s(k)$, s(k) is got from KF. ε(N) is a disturbance vector, and its variance is given by:

(A19) $$\Sigma (N)=\left[\begin{array}{cccc} s(n+1) & 0 & \ldots & 0\\ 0 & s(n+2) & \ldots & 0\\ & & \ddots & \\ 0 & 0 & \cdots & s(n+l) \end{array}\right].$$

From Equation (A18), we can get:

(A20) $$\hat{F}(N)={\left[\psi^{T}(N)\Sigma^{-1}(N)\psi (N)\right]}^{-1}\psi^{T}(N)\Sigma^{-1}(N)Y(N).$$

The error covariance matrix is:

(A21) $$\begin{array}{ll} P_{b}(N) & =E\left\{\left[F-\hat{F}(N)\right]{\left[F-\hat{F}(N)\right]}^{T}\right\}\\ & ={\left[\psi^{T}(N)\Sigma^{-1}(N)\psi (N)\right]}^{-1} \end{array}.$$

Including forgetting factor *γ*, from Equation (A19) we get:

(A22) $$\Sigma^{-1}(N)=\left[\begin{array}{cccc} s^{-1}(n+1)\gamma^{l-1} & 0 & \ldots & 0\\ 0 & s^{-1}(n+2)\gamma^{l-2} & \ldots & 0\\ & & \ddots & \\ 0 & 0 & \cdots & s^{-1}(n+l) \end{array}\right].$$

For *k* = *n* + 1, from Equations (A17), (A18), and (A22), we have:

(A23) $$\bar{Z}(N+1)=B(N+1)F+\hat{Z}(N+1)$$

(A24) Y(N+1)=ψ(N+1)F(N+1)+ε(N+1)

(A25) $$\Sigma^{-1}(N+1)=\left[\begin{array}{cc} \gamma \Sigma^{-1}(N) & 0\\ 0 & s^{-1}(N+1) \end{array}\right],$$

where

$$Y(N+1)=\left[\begin{array}{l} Y(N)\\ \bar{Z}(N+1) \end{array}\right]\begin{array}{ccc} & & \psi (N+1)=\left[\begin{array}{l} \psi (N)\\ B(N+1) \end{array}\right] \end{array}\begin{array}{ccc} & & \varepsilon (N+1)=\left[\begin{array}{l} \varepsilon (N)\\ \hat{Z}(N+1) \end{array}\right] \end{array}.$$

From Equations (A20) and (A21), we have:

(A26) $$\begin{array}{cc} \hat{F}(N+1) & ={\left[\psi^{T}(N+1)\Sigma^{-1}(N+1)\psi (N+1)\right]}^{-1}\psi^{T}(N+1)\Sigma^{-1}(N+1)Y(N+1)\\ & ={\left[\gamma \psi^{T}(N)\Sigma^{-1}(N)\psi (N)+B^{T}(N+1)s^{-1}(N+1)B(N+1)\right]}^{-1}\left[\gamma \psi^{T}(N)\Sigma^{-1}(N)Y(N)+B^{T}(N+1)s^{-1}(N+1)\bar{Z}(N+1)\right] \end{array}$$

(A27) $$\begin{array}{ll} P_{b}(N+1) & ={\left[\psi^{T}(N+1)\Sigma^{-1}(N+1)\psi (N+1)\right]}^{-1}\\ & ={\left[\gamma P_{b}{}^{-1}(N)+B^{T}(N+1)s^{-1}(N+1)B(N+1)\right]}^{-1} \end{array}.$$

Substituting Equation (A21) into Equation (A27), we have:

(A28) $$P_{b}(N+1)=\gamma^{-1}P_{b}(N)-\gamma^{-1}P_{b}(N)B^{T}(N+1){\left[B(N+1)\gamma^{-1}P_{b}(N)B^{T}(N+1)+s(N+1)\right]}^{-1}\begin{array}{c} B(N+1)\gamma^{-1}P_{b}(N) \end{array}$$

Substituting Equation (A20) into Equation (A26), we have:

(A29) $$\begin{array}{cc} \hat{F}(N+1)= & \hat{F}(N)+\gamma^{-1}P_{b}(N)B^{T}(N+1)s^{-1}(N+1)\bar{Z}(N+1)-\gamma^{-1}P_{b}(N)B^{T}(N+1)\times \\ & \left[{\left[B(N+1)\gamma^{-1}P_{b}(N)B^{T}(N+1)+s(N+1)\right]}^{-1}B(N+1)\right]\left[\hat{F}(N)+\gamma^{-1}P_{b}(N)B^{T}(N+1)s^{-1}(N+1)\bar{Z}(N+1)\right] \end{array}$$

We insert the following term between $B^{T}(N+1)$ and $s^{-1}(N+1)$, and get the following outcome:

$${\left[B(N+1)\gamma^{-1}P_{b}(N)B^{T}(N+1)+s(N+1)\right]}^{-1}\begin{array}{cc} \times & \left[B(N+1)\gamma^{-1}P_{b}(N)B^{T}(N+1)+s(N+1)\right] \end{array}.$$

## Appendix B

Following the nonlinear discrete system is given:

(A30) $$X_{k}=f(X_{k-1},F_{k-1})+w_{k}$$

(A31) $$Z_{k}=h(X_{k})+v_{k}.$$

The posteriori state estimate without exciting force:

(A32) $$\begin{array}{ll} {\bar{X}}_{k} & =f({\bar{X}}_{k-1})+K_{k}[Z_{k}-h(f({\bar{X}}_{k-1}))]\\ & =f({\bar{X}}_{k-1})-K_{k}h(f({\bar{X}}_{k-1}))+K_{k}Z_{k} \end{array}.$$

The posteriori state estimate with exciting force:

(A33) $$\begin{array}{ll} {\hat{X}}_{k} & =f({\hat{X}}_{k-1},F_{k-1})+K_{k}[Z_{k}-h(f({\hat{X}}_{k-1},F_{k-1}))]\\ & =f({\hat{X}}_{k-1},F_{k-1})-K_{k}h(f({\hat{X}}_{k-1},F_{k-1}))+K_{k}Z_{k}\\ & =f({\hat{X}}_{k-1},0)+\Gamma_{k}F_{k-1}+K_{k}Z_{k}-K_{k}h(f({\hat{X}}_{k-1},0)+\Gamma_{k}F_{k-1})\\ & =f({\hat{X}}_{k-1},0)+\Gamma_{k}F_{k-1}+K_{k}Z_{k}-K_{k}(h(f({\hat{X}}_{k-1},0))+\Phi_{k}\Gamma_{k}F_{k-1}) \end{array},$$

where $K_{k}$ is obtained from CKF, and

$$\Phi_{k}=\partial f(\hat{X}(k|k-1))/\partial X\begin{array}{cc} & \Gamma_{k}=\partial f(\hat{X}(k|k-1))/\partial F\begin{array}{cc} & H_{k}=\partial h(\hat{X}(k|k-1))/\partial X \end{array} \end{array}.$$

Define the difference of the two posteriori state estimates as follows:

(A34) $$\begin{array}{ll} \Delta X_{k} & ={\hat{X}}_{k}-{\bar{X}}_{k}\\ & =(I-K_{k}H_{k})\Phi_{k}({\hat{X}}_{k-1}-{\bar{X}}_{k-1})+(I-K_{k}H_{k})\Gamma_{k}F_{k-1} \end{array}.$$

Assuming the exciting force begins with time $t_{n}$, then:

1. *k* < *n*. ${\hat{X}}_{k-1}-{\bar{X}}_{k-1}$ = 0. $F_{k-1}$ = 0, so $\Delta X_{k}$ = 0
2. *k* = *n*. ${\hat{X}}_{k-1}-{\bar{X}}_{k-1}$ = 0. $F_{k-1}$ = 0, so $\Delta X_{k}$ = 0
3. *k* > *n*. ${\hat{X}}_{k-1}-{\bar{X}}_{k-1}=\Delta X_{k-1}$, so
  (A35) $$\begin{array}{ll} \Delta X_{k} & =(I-K_{k}H_{k})\Phi_{k}({\hat{X}}_{k-1}-{\bar{X}}_{k-1})+(I-K_{k}H_{k})\Gamma_{k}F_{k-1}\\ & =(I-K_{k}H_{k})(\Phi_{k}\Delta X_{k-1}+\Gamma_{k}F_{k-1}) \end{array}.$$

In summary, we get:

(A36) $$\Delta X_{k}=\left\{ \begin{array}{ll} 0 & k\leq n\\ \left(I-K_{k}H_{k})(\Phi_{k}\Delta X_{k-1}+\Gamma_{k}F_{k-1}\right) & k>n \end{array}\right..$$

At time $t_{n+1}$, Equation (A36) becomes:

(A37) $$\Delta X_{n+1}=(I-K_{n+1}H_{n+1}){\left(\Phi_{n+1}\Delta X_{n}+\Gamma_{n+1}F_{n}\right)}_{}.$$

From Equation (A36) we know that $\Delta X_{n}$ = 0, so Equation (A37) becomes:

(A38) $$\Delta X_{n+1}=(I-K_{n+1}H_{n+1})\Gamma_{n+1}F_{n}.$$

Define

$$M_{n+1}=I-K_{n+1}H_{n+1}.$$

Then Equation (A38) becomes:

(A39) $$\Delta X_{n+1}=M_{n+1}\Gamma_{n+1}F_{n}.$$

From Equations (A36) and (A39), for *k* > *n*, we have:

(A40) $$\Delta X_{k}=(I-K_{k}H_{k})(\Phi_{k}\Delta X_{k-1}+\Gamma_{k}F_{k-1}).$$

Ignoring $\Phi_{k}\Delta X_{k-1}$, Equation (A40) becomes:

(A41) $$\Delta X_{k}=M_{k}\Gamma_{k}F_{k-1}.$$

From Equations (A41) and (A40), we have:

$$\begin{array}{ll} M_{k}\Gamma_{k}F_{k-1} & =(I-K_{k}H_{k})(\Phi_{k}\Delta X_{k-1}+\Gamma_{k}F_{k-1})\\ & =(I-K_{k}H_{k})\Phi_{k}M_{k-1}\Gamma_{k-1}F_{k-2}+(I-K_{k}H_{k})\Gamma_{k}F_{k-1} \end{array}.$$

Assume $F_{k-1}$ = $F_{k-2}$, then:

(A42) $$M_{k}=(I-K_{k}H_{k})(\Phi_{k}M_{k-1}+I).$$

From Equations (A41) and (A42), we have:

(A43) $${\hat{X}}_{k}={\bar{X}}_{k}+M_{k}\Gamma_{k}F_{k-1}{}_{},$$

where:

$$M_{k}=\left\{ \begin{array}{ll} 0 & k\leq n\\ \left(I-K_{k}H_{k})(\Phi_{k}M_{k-1}+I\right) & k>n \end{array}\right..$$

The observed value of the residual sequence with exciting force can be described as:

(A44) $${\hat{Z}}_{k}=Z_{k}-h(f({\hat{X}}_{k-1},F_{k-1})).$$

The observed value of the residual sequence without exciting force can be described as:

(A45) $${\bar{Z}}_{k}=Z_{k}-h(f({\bar{X}}_{k-1})).$$

For different values of *k*, we have:

1. *k* < *n*. $F_{k-1}$ = 0, ${\bar{Z}}_{k}={\hat{Z}}_{k}$
2. *k* = *n*. $F_{k-1}$ = 0, ${\bar{Z}}_{k}={\hat{Z}}_{k}$
3. *k* > *n*. $F_{k-1}\neq 0\begin{array}{cc} , & {\bar{Z}}_{k}\neq {\hat{Z}}_{k} \end{array}$
  $$\begin{array}{ll} {\bar{Z}}_{k}-{\hat{Z}}_{k} & =H_{k}\Phi_{k}({\hat{X}}_{k-1}-{\bar{X}}_{k-1})+H_{k}\Gamma_{k}F\\ & =H_{k}(\Phi_{k}M_{k-1}+I)\Gamma_{k}F \end{array}.$$

In summary, we get:

(A46) $${\bar{Z}}_{k}=\left\{ \begin{array}{ll} {\hat{Z}}_{k} & k\leq n\\ {\hat{Z}}_{k}+B_{k}F & k>n \end{array}\right.,$$

where:

$$B_{k}=H_{k}(\Phi_{k}M_{k-1}+I)\Gamma_{k}.$$

For *k* = *n* + 1,*n* + 2, ... *n* + *l*. we have:

(A47) Y=ψF+ε,

where:

$$Y(N)=\left[\begin{array}{l} {\bar{Z}}_{n+1}\\ {\bar{Z}}_{n+2}\\ \vdots \\ {\bar{Z}}_{n+l} \end{array}\right]\begin{array}{ccc} & & \varepsilon (N)= \end{array}\left[\begin{array}{l} {\hat{Z}}_{n+1}\\ {\hat{Z}}_{n+2}\\ \vdots \\ {\hat{Z}}_{n+l} \end{array}\right]\begin{array}{ccc} & & \psi (N)=\left[\begin{array}{l} B(n+1)\\ B(n+2)\\ \vdots \\ B(n+l) \end{array}\right]=\left[\begin{array}{l} H_{n+1}\Gamma_{n+1}\\ H_{n+2}(\Phi_{n+2}M_{n+1}+I)\Gamma_{n+2}\\ \vdots \\ H_{n+l}(\Phi_{n+l}M_{n+l-1}+I)\Gamma_{n+l} \end{array}\right] \end{array}$$

$$M_{n+l}=\left\{ \begin{array}{ll} 0 & l=0\\ \left(I-K_{n+l}H_{n+l})[\Phi_{n+l}M_{n+l-1}+I\right] & l>0 \end{array}.\right.$$

Assume $E[\hat{z}(k){\hat{z}}^{T}(k)]=s(k)$, s(k) is got from CKF. ε(N) is a disturbance vector, and its variance is given by:

(A48) $$\Sigma (N)=\left[\begin{array}{cccc} s(n+1) & 0 & \ldots & 0\\ 0 & s(n+2) & \ldots & 0\\ & & \ddots & \\ 0 & 0 & \cdots & s(n+l) \end{array}\right].$$

From Equation (A47), we can get:

(A49) $$\hat{F}(N)={\left[\psi^{T}(N)\Sigma^{-1}(N)\psi (N)\right]}^{-1}\psi^{T}(N)\Sigma^{-1}(N)Y(N).$$

The error covariance matrix is:

(A50) $$\begin{array}{ll} P_{b}(N) & =E\left\{\left[F-\hat{F}(N)\right]{\left[F-\hat{F}(N)\right]}^{T}\right\}\\ & ={\left[\psi^{T}(N)\Sigma^{-1}(N)\psi (N)\right]}^{-1} \end{array}.$$

Including forgetting factor *γ*, from (A48), we get:

(A51) $$\Sigma^{-1}(N)=\left[\begin{array}{cccc} s^{-1}(n+1)\gamma^{l-1} & 0 & \ldots & 0\\ 0 & s^{-1}(n+2)\gamma^{l-2} & \ldots & 0\\ & & \ddots & \\ 0 & 0 & \cdots & s^{-1}(n+l) \end{array}\right].$$

For *k* = *n* + 1, from Equations (A46), (A47), and (A51), we have:

(A52) $$\bar{Z}(N+1)=B(N+1)F+\hat{Z}(N+1)$$

(A53) Y(N+1)=ψ(N+1)F(N+1)+ε(N+1)

(A54) $$\Sigma^{-1}(N+1)=\left[\begin{array}{cc} \gamma \Sigma^{-1}(N) & 0\\ 0 & s^{-1}(N+1) \end{array}\right],$$

where

$$Y(N+1)=\left[\begin{array}{l} Y(N)\\ \bar{Z}(N+1) \end{array}\right]\begin{array}{ccc} & & \psi (N+1)=\left[\begin{array}{l} \psi (N)\\ B(N+1) \end{array}\right] \end{array}\begin{array}{ccc} & & \varepsilon (N+1)=\left[\begin{array}{l} \varepsilon (N)\\ \hat{Z}(N+1) \end{array}\right] \end{array}.$$

From Equations (A49) and (A50), we have:

(A55) $$\begin{array}{cc} \hat{F}(N+1) & ={\left[\psi^{T}(N+1)\Sigma^{-1}(N+1)\psi (N+1)\right]}^{-1}\psi^{T}(N+1)\Sigma^{-1}(N+1)Y(N+1)\\ & ={\left[\gamma \psi^{T}(N)\Sigma^{-1}(N)\psi (N)+B^{T}(N+1)s^{-1}(N+1)B(N+1)\right]}^{-1}\left[\gamma \psi^{T}(N)\Sigma^{-1}(N)Y(N)+B^{T}(N+1)s^{-1}(N+1)\bar{Z}(N+1)\right] \end{array}$$

(A56) $$\begin{array}{ll} P_{b}(N+1) & ={\left[\psi^{T}(N+1)\Sigma^{-1}(N+1)\psi (N+1)\right]}^{-1}\\ & ={\left[\gamma P_{b}{}^{-1}(N)+B^{T}(N+1)s^{-1}(N+1)B(N+1)\right]}^{-1} \end{array}.$$

Substituting Equation (A50) into Equation (A56), we have:

(A57) $$P_{b}(N+1)=\gamma^{-1}P_{b}(N)-\gamma^{-1}P_{b}(N)B^{T}(N+1){\left[B(N+1)\gamma^{-1}P_{b}(N)B^{T}(N+1)+s(N+1)\right]}^{-1}\begin{array}{c} B(N+1)\gamma^{-1}P_{b}(N) \end{array}$$

Substituting Equation (A49) into Equation (A55), we have:

(A58) $$\begin{array}{cc} \hat{F}(N+1)= & \hat{F}(N)+\gamma^{-1}P_{b}(N)B^{T}(N+1)s^{-1}(N+1)\bar{Z}(N+1)-\gamma^{-1}P_{b}(N)B^{T}(N+1)\times \\ & \left[{\left[B(N+1)\gamma^{-1}P_{b}(N)B^{T}(N+1)+s(N+1)\right]}^{-1}B(N+1)\right]\left[\hat{F}(N)+\gamma^{-1}P_{b}(N)B^{T}(N+1)s^{-1}(N+1)\bar{Z}(N+1)\right] \end{array}.$$

We insert the following term between $B^{T}(N+1)$ and $s^{-1}(N+1)$, and get the following outcome:

$${\left[B(N+1)\gamma^{-1}P_{b}(N)B^{T}(N+1)+s(N+1)\right]}^{-1}\begin{array}{cc} \times & \left[B(N+1)\gamma^{-1}P_{b}(N)B^{T}(N+1)+s(N+1)\right] \end{array}.$$
